# High performance MRI simulations of motion on multi-GPU systems

**DOI:** 10.1186/1532-429X-16-48

**Published:** 2014-07-04

**Authors:** Christos G Xanthis, Ioannis E Venetis, Anthony H Aletras

**Affiliations:** 1 Department of Computer Science and Biomedical Informatics, University of Thessaly, Lamia, Greece; 2 Department of Clinical Physiology, Skåne University Hospital Lund, Lund University, Lund, Sweden; 3 Department of Computer Engineering and Informatics, University of Patras, Patras, Greece

## Abstract

**Background:**

MRI physics simulators have been developed in the past for optimizing imaging protocols and for training purposes. However, these simulators have only addressed motion within a limited scope. The purpose of this study was the incorporation of realistic motion, such as cardiac motion, respiratory motion and flow, within MRI simulations in a high performance multi-GPU environment.

**Methods:**

Three different motion models were introduced in the Magnetic Resonance Imaging SIMULator (MRISIMUL) of this study: cardiac motion, respiratory motion and flow. Simulation of a simple Gradient Echo pulse sequence and a CINE pulse sequence on the corresponding anatomical model was performed. Myocardial tagging was also investigated. In pulse sequence design, software crushers were introduced to accommodate the long execution times in order to avoid spurious echoes formation.

The displacement of the anatomical model isochromats was calculated within the Graphics Processing Unit (GPU) kernel for every timestep of the pulse sequence. Experiments that would allow simulation of custom anatomical and motion models were also performed. Last, simulations of motion with MRISIMUL on single-node and multi-node multi-GPU systems were examined.

**Results:**

Gradient Echo and CINE images of the three motion models were produced and motion-related artifacts were demonstrated. The temporal evolution of the contractility of the heart was presented through the application of myocardial tagging. Better simulation performance and image quality were presented through the introduction of software crushers without the need to further increase the computational load and GPU resources. Last, MRISIMUL demonstrated an almost linear scalable performance with the increasing number of available GPU cards, in both single-node and multi-node multi-GPU computer systems.

**Conclusions:**

MRISIMUL is the first MR physics simulator to have implemented motion with a 3D large computational load on a single computer multi-GPU configuration. The incorporation of realistic motion models, such as cardiac motion, respiratory motion and flow may benefit the design and optimization of existing or new MR pulse sequences, protocols and algorithms, which examine motion related MR applications.

## Background

Since the introduction of the first nuclear magnetic resonance physics simulator by Summers, Axel and Israel in 1986 [1], several magnetic resonance imaging (MRI) physics simulators have been developed to serve different purposes, such as training of physicists and technologists, optimization of imaging protocols, answering methodological issues and identification of particular artifact sources [2-5]. The use of Bloch equation simulators has been shown to be especially useful for answering specific methodological problems, such as T1 mapping accuracy and precision [6-8]. In contrast to such targeted applications of Bloch equation simulators, multi-use advanced simulation platforms had been also presented in the literature [9].

In MRI, motion is one major source of artifacts, which may degrade image quality. Such artifacts are generated from rhythmic motion such as blood flow, respiration, cardiac motion, and, sometimes, from unpredictable patient body motion while MRI data are acquired. In many cases, even a small amount of motion may induce large errors in some MR-related applications, such as in functional magnetic resonance imaging (fMRI) analysis [10]. Image acquisition becomes more challenging in cardiac MR applications due to heart motion caused by both the beating heart during the cardiac cycle as well as by its motion as a result of the respiratory cycle [11].

The optimization of MRI pulse sequences and imaging protocols may involve techniques and algorithms that try to detect and minimize motion artifacts, which may contaminate the acquired magnetic resonance (MR) signal. However, these techniques may be time consuming and/or may involve human volunteer experimentation, animal models or the development of advanced phantoms to simulate physiological motion [12-14]. MR physics simulations of motion can also help towards optimizing and developing pulse sequences and imaging protocols. Such simulations not only can be used for investigating motion artifact sources and developing new motion compensation techniques but also for training purposes.

To date, few MR simulators that incorporate simulation of motion and motion artifacts have been proposed. Petersson et al. [15] developed a simulation method based on k-space formalism. Although this simulator simulates movement or flow by allowing signal phase changes during the sampling procedure, it does not allow for the simulation of more realistic experiments. POSSUM [10] is a more advanced simulation platform which focuses on fMRI and incorporates realistic rigid-body motion of the head anatomical object. JEMRIS [16] is the most disseminated advanced simulator platform to date. It is based on numerical solutions of the Bloch equations and has incorporated motion of custom anatomical objects.

Although both POSSUM and JEMRIS were designed and developed taking into account the simulation of spin motion, they have incorporated several assumptions to their simulations. POSSUM is limited to applications relevant to fMRI. Both POSSUM and JEMRIS usually simulate spins only within the slice of interest and not within the entire 3D anatomical object. However, this assumption is not related to any specific design limitation of these simulation platforms but rather imposed due to the high computational load introduced by the simulation of 3D motion during the entire course of the pulse sequence. Even though both these simulation platforms can achieve shorter execution times by parallelizing simulations on computer systems of multiple nodes, this requires advanced computer cluster setups and advanced technical knowledge. Moreover, both POSSUM and JEMRIS do not incorporate cardiac motion, respiratory motion or other more complicated forms of motion originating from the patient.

High performance low-cost parallel computing was recently introduced in MR simulations. The recently developed Magnetic Resonance Imaging SIMULator (MRISIMUL) is an advanced GPU-based (Graphics Processing Units), step-by-step comprehensive Bloch equation simulation platform of MR physics that allows for application in large scale analysis without model simplifications [17]. Previous results on a single board GPU personal computer [17] demonstrated high computational speedup of GPU-based simulations when compared to CPU-based and OpenMP-based simulations with multithreading. However, to date, despite the implementation of simulations on a fast GPU-based system, realistic motion simulations have not yet been demonstrated in MRI.

The specific aim of this study was the incorporation of realistic motion in a high performance multi-GPU environment of MRI simulations. We hypothesized that cardiac motion, respiratory motion and flow during the entire course of the MR pulse sequence could be simulated within a reasonable amount of time by distributing the computational load on a multi-GPU multi-node system. We hypothesized that an MR simulator could be designed and implemented around a user-defined motion model for simulation of MRI physics within the entire space occupied by a three dimensional anatomical object.

## Methods

MRISIMUL utilized CUDA technology (NVIDIA, Santa Clara, CA) and a simulation wrapper developed in MATLAB (The Mathworks, Inc., Natick, MA). MATLAB handled the development of the 3D anatomical object, the programming of the pulse sequence, the reconstruction of the final image and other MR imaging processes such as the development of inhomogeneity and RF sensitivity maps. The computationally demanding core services (kernel) of MRISIMUL were developed in CUDA-C and distributed in parallel within the graphic processing unit (GPU). The kernel handled the multiplication and summation of large matrices for every spatial point of the anatomical object for every time point of the pulse sequence.

As described in previous work [17], one of the inputs to the MRISIMUL kernel was the anatomical object. The anatomical object consisted of isochromats (i.e. small groups of spins which shared the same characteristics) as shown in the following notation:

(1) $$O\left(\vec{r},\mathrm{tissue}\right)=\left\{\begin{array}{c} r_{x},r_{y},r_{z},T_{1}\left(\mathrm{tissue}\right),T_{2}\left(\mathrm{tissue}\right),\\ \rho \left(\mathrm{tissue}\right),\omega \left(\mathrm{tissue}\right) \end{array}\right\}$$

where $\vec{r}=\left(r_{x},r_{y},r_{z}\right)$ defined the spatial coordinates, T1, T2 and ρ defined the spin–lattice relaxation time, spin-spin relaxation time and proton density of the corresponding tissue respectively and ω was the precession frequency of the corresponding tissue within the main magnetic field. The time evolution of the isochromat magnetization vector was described by the following equation:

(2) $$\vec{M}\left(\vec{r},t+\mathrm{\Delta t}\right)=\vec{A}\left(\mathrm{\Delta t}\right)+B\left(\vec{r},t,\mathrm{\Delta t}\right)\cdot \vec{M}\left(\vec{r},t\right)$$

where Δt defined the time step of the pulse sequence, A(t) described the component of the T1 relaxation towards the equilibrium state that the magnetization vector of the isochromat had at the start of the pulse sequence and B(t) described the accumulated effect of isochromat relaxations, pulse sequence pulses (RF, gradients) and magnetic field inhomogeneity on the magnetization vector of the isochromat [17].

### Motion models

The introduction of motion involved the update of spatial coordinates of the isochromats for every single time step of the pulse sequence. Equation (1) was modified according to

(3) $$O\left(\vec{r\left(t\right)},\mathrm{tissue}\right)=\left\{\begin{array}{l} r_{x}\left(t\right),r_{y}\left(t\right),r_{z}\left(t\right),T_{1}\left(\mathrm{tissue}\right),\\ T_{2}\left(\mathrm{tissue}\right),\rho \left(\mathrm{tissue}\right),\omega \left(\mathrm{tissue}\right) \end{array}\right\}$$

and equation (2) was updated according to the following equation:

(4) $$\vec{M}\left(\vec{r\left(t+\mathrm{\Delta t}\right)},t+\mathrm{\Delta t}\right)=\vec{A}\left(\mathrm{\Delta t}\right)+B\left(\vec{r\left(t\right)},t,\mathrm{\Delta t}\right)\cdot \vec{M}\left(\vec{r\left(t\right)},t\right)$$

The time dependence of the isochromat spatial coordinates allowed for the introduction of a motion model for all isochromats within the anatomical object. In this work, three different motion models were examined: respiratory motion, heart motion and blood flow.

a. Respiratory motion

Initially, respiratory motion of the diaphragm was simulated. Previous studies [18,19] have demonstrated that the motion of the diaphragm due to breathing in most cases can be described by periodicity and asymmetry. In this study, we applied the respiratory model described by Lujan et al. [20], which approximates the position of the diaphragm as a function of time based on

b. Heart motion

For simulating heart motion, a mathematical model of myocardial deformation was applied on a cylinder [21,22]. This model accounted for longitudinal and radial contraction, axial torsion and rigid body displacement. The displacement in every point of the cylinder was computed by the following analytical expression in cylindrical coordinates [21]:

c. Blood flow

Laminar flow of a homogeneous liquid within a straight tube was simulated. The displacement in every point of the liquid was simulated along one axis only and it was expressed as a function of time by the following equation:

(5) $$z\left(t\right)=z_{0}-b\;{\left(\cos\left(\frac{\mathrm{\pi t}}{T}-\varphi \right)\right)}^{2n}$$

where z_0_ was the position of diaphragm during end exhalation, z_0_-b was the position of diaphragm during end inhalation, T was the period of the breathing cycle, n defined the steepness and flatness of the motion model and *φ* defined the initial phase of the breathing cycle.

(6) $$r=\;\sqrt{{r_{i}}^{2}+\frac{\left(R^{2}-R_{i}^{2}\right)}{\lambda }}$$

(7) θ=φR+Θ+γΖ+ϵ

(8) z=ωR+λΖ+δ

where (*r*, *θ*, *z*) described the position of tissue in the deformed state and (*R*, Θ, Ζ) its position prior to deformation, R_i_ and r_i_ were the inner radii of the cylinder before and after the deformation respectively, *λ* described the longitudinal contraction, *φ*  described the R-Θ shear, *γ*  described the axial torsion, *ϵ*  described the rigid body rotation, ω described the R-Z shear and δ the rigid body displacement.

(9) $$z\left(t\right)=z_{0}+\mathrm{vt}$$

where *z*_0_  described the initial position of the point and *v* described its velocity according to the velocity profile for laminar flow. The velocity profile of laminar flow within the tube as a function of the radius r was given by:

(10) $$v\left(r\right)=v_{\max}\left(1-\frac{r^{2}}{R^{2}}\right)$$

where *v*_*max*_ was the maximum velocity of the liquid at the center of the tube and R was the inner radius of the tube.

### Implementation

In brief, for single node multi-GPU simulations, three different computer generated anatomical models were used in this study, one for each type of motion described in the previous section. For each type of motion, simulation of a simple Gradient Echo (GE) pulse sequence and a CINE pulse sequence on the corresponding anatomical model was performed. The displacement of the anatomical model isochromats was calculated within the CUDA-C kernel for every timestep of the pulse sequence. In other experiments, calculation of the displacements within the MATLAB wrapper was also performed. In these experiments the goal was to examine the output and the execution time of simulations that would allow for experiments of custom anatomical and motion models. Last, MRI simulations of motion with MRISIMUL were tested on multi-node multi-GPU computer systems in order to allow for even faster execution times for more complex MR simulations.

a. Simulator design

Prior to executing kernel computations within the CUDA environment, the entire pulse sequence was transferred to the global memory of the GPU. The pulse sequence was represented as a matrix of 6 row vectors. The first five row vectors handled the time evolution of the pulse sequence events, that is, the RF excitation on x and y axis and the gradients amplitude along the three axes. The sixth row represented the off/on state (0 or 1 respectively) of the RF receiver. Along with the pulse sequence, the matrix holding the MR characteristics of the different tissues within the 3D anatomical model (i.e. T1 and T2 relaxation times, proton density and chemical shift) was also transferred to the global memory of the GPU.

Simulations executed on a single GPU board, the anatomical model was divided evenly in space and the kernel was called for every part of the model. For every kernel call, three matrices were transferred to the global memory of the GPU. These matrices held the spatial coordinates, the magnetization vector components and the type of the corresponding tissue of the isochromats within the specific part of the 3D object. In some cases, depending on the type of motion being simulated, additional matrices were transferred to the global memory of the GPU board. In particular, for flow simulations, a matrix containing the velocities of the isochromats within the specific part of the object, based on equation (10), was transferred to the GPU. For heart motion, the matrices containing the spatial coordinates of both the undeformed and deformed states of the heart model were transferred to the global memory of the GPU.

A different kernel was used for each case depending on the type of motion. A different piece of the 3D anatomical model was transferred to the GPU for every kernel call. For each kernel call, the new spatial coordinates, due to motion, of the isochromats were calculated in parallel. The magnetization vectors were computed for every time step of the pulse sequence. Summation of the magnetization vectors was performed for time steps when the RF receiver was on and then saved in a matrix within the global memory. This k-space matrix of the piece of the 3D anatomical model was transferred back to the host at the end of every kernel call. It was subsequently summed with the matrices corresponding to other pieces of the 3D anatomical model to yield the total k-space matrix. The image was then reconstructed by 2D Fourier transformation of the total k-space matrix.

b. Motion models

**Figure 1:**
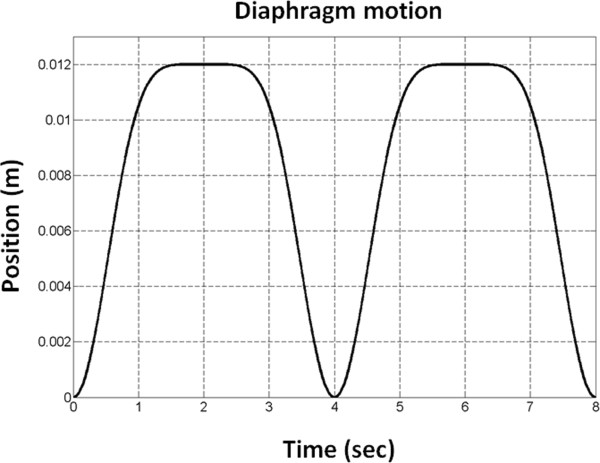
**Respiratory model simulations.** Illustration of the temporal evolution of the position of the diaphragm for two consecutive breathing cycles.

For respiratory motion simulations, a homogeneous 3D user-defined phantom was used. The size of this 3D model was of 10 cm × 2.5 cm × 20 cm. The isochromat volume size was 0.5 mm × 0.5 mm × 1 mm resulting in 2000000 isochromats within the object. The object was placed in space and translational motion was induced on the y-axis (Phase Encoding axis) only, according to equation 5. In this motion model, z_0_ and b were set equal to 0.012 m, φ was set to 0 and n to 3. According to Lujan et al. [20] these values represent an average observed diaphragm motion. A period T of 4 sec (respiratory rate = 15 breaths per minute) was selected for the breathing cycle. The position of the diaphragm is illustrated in Figure 1.

The computer model used for heart motion simulations was a homogeneous cylindrical 3D user-defined object. The size of the cylinder was 100 mm × 100 mm × 80 mm, the inner diameter was 50 mm and the isochromat volume size was 0.25 mm × 0.25 mm × 1 mm. Based on this configuration, the total number of isochromats was 7632792. T1 and T2 relaxation times were set to 0.9 sec and 0.05 sec respectively. The deformation of the cylinder followed equations (6–8) where the values of model parameters were chosen so as to represent human heart motion, according to Tecelao et al. [21]. In detail, the model parameters were set: *λ* = 1, φ = 0.556, γ = 0.6, ϵ = 18.334, ω = 0.278 and δ = 4.167.

**Figure 2:**
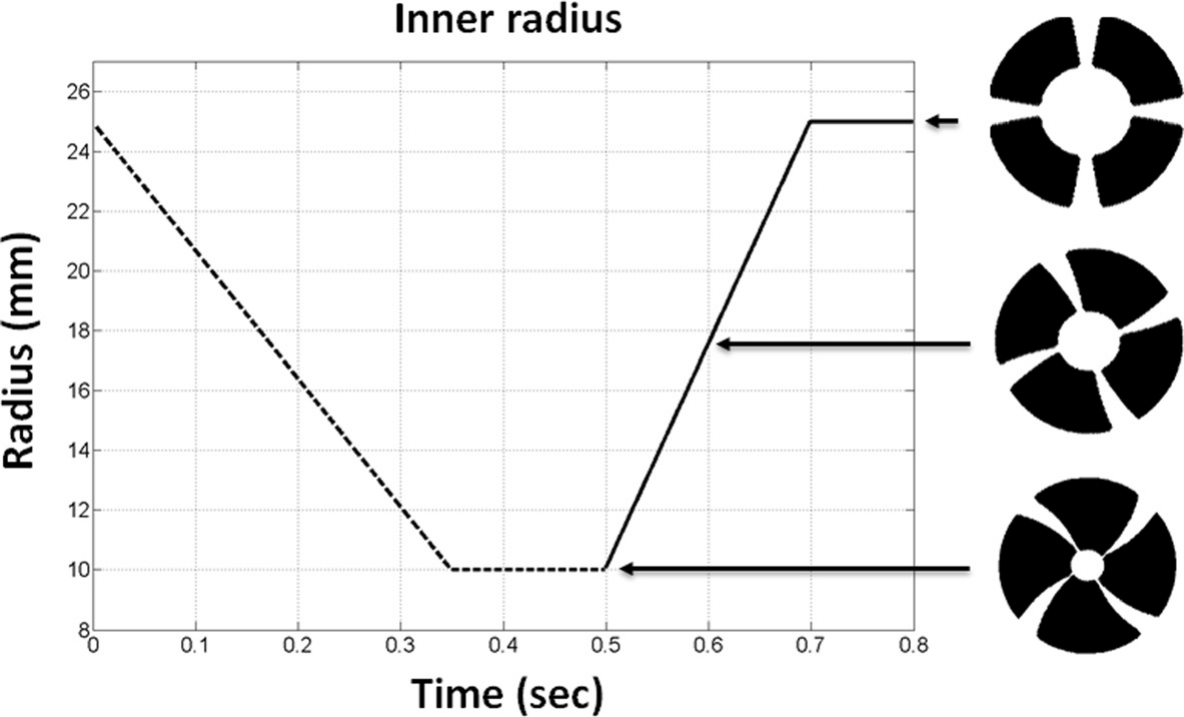
**Cardiac model simulations: plot of the temporal evolution of the inner radius of the heart motion model.** Systole is shown with the dashed line whereas diastole is shown with the solid line. The corresponding phases of the heart model are shown on the right. The artificial radial white line tags have been placed intentionally to demonstrate the deformation of the heart model throughout the cardiac cycle.

The cylinder of the myocardial model was initialized in the undeformed state (i.e. simulating cardiac end diastole). A linear interpolation of the cylindrical coordinates between the initial and the deformed state was performed for every timestep of the cardiac cycle. Figure 2 shows the time evolution for an inner radius R_i_ of 25 mm in the undeformed cylinder and an inner radius r_i_ of 10 mm in the deformed cylinder (total duration 0.8 sec).

**Figure 3:**
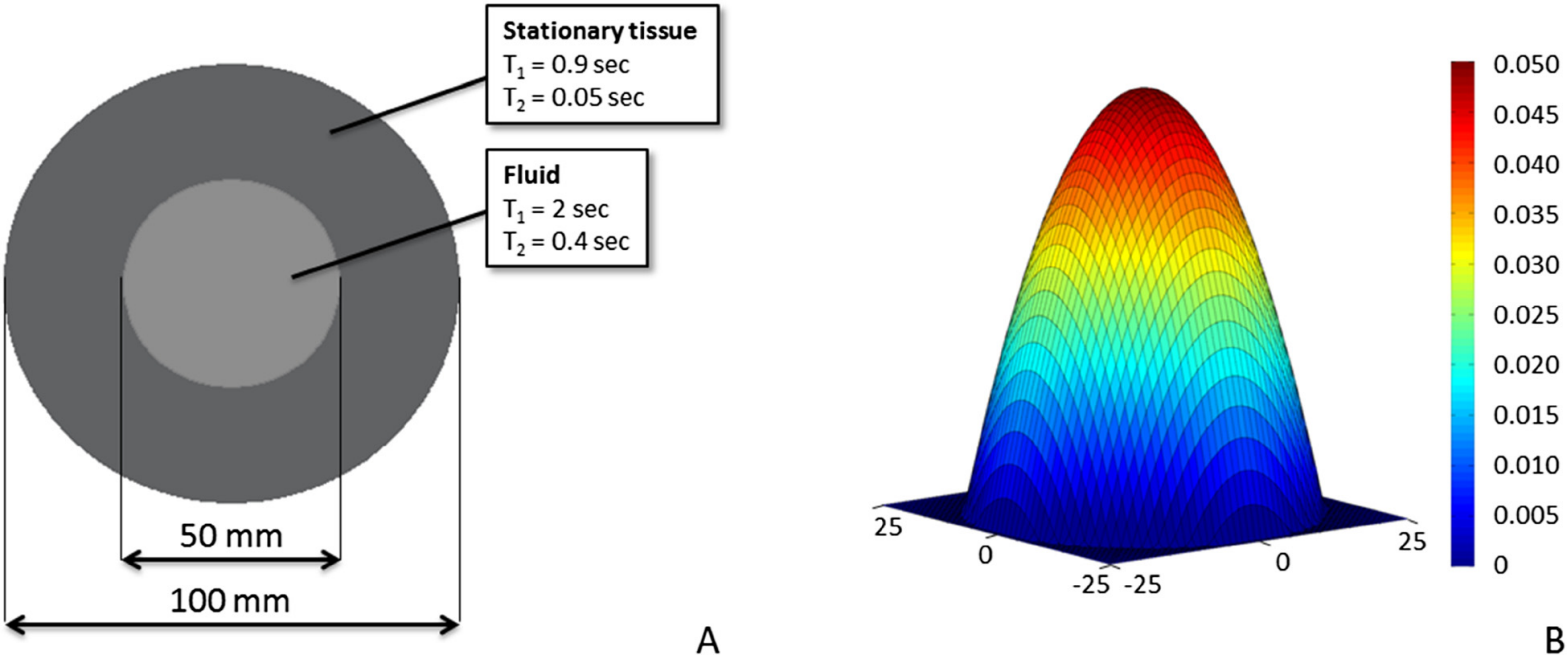
**Flow simulations. (A)** Illustration of the geometry of the flow anatomical model. The outer cylinder represents stationary tissue whereas the inner cylinder represents the fluid. **(B)** Laminar velocity profile of the fluid within the cylindrical flow model.

Last, for flow simulations, a custom 3D object of two cylinders, one inside the other, was used (Figure 3A). In this anatomical model, the outer cylinder had a diameter of 10 cm and length of 1 m whereas the inner cylinder had a diameter of 5 cm and length also 1 m. The outer cylinder was stationary whereas the inner cylinder represented fluid with particles flowing along the longitudinal axis of the cylinder with a fixed velocity profile given by equation (10). The maximum velocity of the liquid at the center of the inner cylinder was set to 0.05 m/sec, which is within the range of aortic blood flow velocities during cardiac ejection [23]. The 3D velocity profile of laminar flow is illustrated in Figure 3B. The isochromat volume size was 0.25 mm × 0.25 mm × 1 mm resulting in 30779693 isochromats. The outer cylinder had relaxation times T1 and T2 of 0.9 sec and 0.05 sec respectively. The corresponding values of the inner cylinder were set to 2 sec and 0.4 sec respectively.

c. Pulse sequences

A Gradient Echo (GE) pulse sequence was applied to all three anatomical models with and without motion. A segmented CINE pulse sequence was applied on respiratory and heart models with motion. For both pulse sequences, the static magnetic field strength was set to 1.5 Tesla, the temporal resolution of the simulator was set to 10 μsec, the bandwidth of the receiver was 50 kHz, the slice thickness was 10 mm and the k-space matrix was 256 × 256. The spin system was brought to steady state by means of dummy excitations prior to recording any signal. For respiratory motion simulations the FOV was set to 150 mm × 150 mm whereas for heart motion simulations it was set to 360 mm × 270 mm.

The GE pulse sequence was applied with a three lobe sinc-shaped RF pulse of 15° and 2 msec duration and a TE/TR of 4.5/50 msec. Gradient crushers were introduced at the end of each TR (see next section). For flow model simulations a bipolar velocity-encoding (venc) gradient pair was introduced after the RF excitation pulse. The bipolar gradient was applied along the slice direction with a venc of 10 cm/sec. The velocity encoded GE pulse sequence was run twice with different polarity of the venc gradient pair. The velocity encoded phase image was computed from the two complex images.

In the cases of respiratory and heart motion simulations, the CINE pulse sequence was based on the same design as the GE pulse sequence with a flip angle of 40° and TR of 8 msec. The acquisition was segmented along the respiratory and cardiac cycles with different combinations of phases per cycle and views per segment (vps). For the respiratory model simulation, 250 phases and 2 vps, 125 phases and 4 vps and, last, 100 phases and 5 vps were tested. For the heart model simulation 100 phases and 1 vps, 50 phases and 2 vps and, last, 20 phases and 5 vps were tested.

Last, myocardial tagging was also investigated. For this purpose, spatial modulation of magnetization (1–1 SPAMM) [24] was introduced upon detection of the R-wave in the CINE pulse sequence with 100 phases and 1 vps. The two SPAMM RF pulses were three-lobe sinc-shaped with a flip angle of 45°, 3 msec duration. The gradient pulse was a rectangular-shaped gradient pulse of 0.6 msec duration and 0.004 T/m strength. Gradient crushers were introduced at the end of the 1–1 SPAMM preparation (see next section).

d. Pulse sequence design and computational demands

As described in previous studies [17,25], the imaging gradients may induce a phase difference of more than 180° among neighboring isochromats within the anatomical object, which in turn may produce spurious nonrealistic echo refocusing. Based on the design of the pulse sequence, MRISIMUL set the maximum isochromat size so that there was no image quality degradation due to the introduction of strong crushers into the pulse sequence. Such strong gradients result in artificial spurious echoes and degradation of the final image quality due to low isochromat density. Avoidance of this effect required decreasing the isochromat size within the anatomical object. This in turn increased the computational load of the simulation and resulted in long execution times. These execution times could become prohibitively long for some simulations (e.g. CINE pulse sequences). The maximum volume size of the isochromats may also need to be further decreased as a result of myocardial strain, which introduces a variable isochromat density over time.

For this purpose, the simulator allowed the introduction of another form of crushers, namely “software crushers”. In this case, the software crushers involved the transfer to the GPU card of an extra matrix consisting of 0 s and 1 s pointing out when the kernel should induce nullification of the transverse components of every isochromat within the anatomical object. The introduction of software crushers may result in not allowing the appearance of some realistic artifacts and contrast (e.g. stimulated echo based artifacts and contrast). However, software crushers allow for larger isochromats volumes and reduced computational load and, therefore, reduced GPU resources. This allows for the execution of otherwise prohibitively long simulations. In any case, it is up to the MRISIMUL user to decide whether to compromise Stimulated Echo artifacts and contrast for faster execution times.

To demonstrate the effects on image quality and computational demands of realistic crushers (which impart phase to the isochromats) against software crushers, four test cases were considered with a CINE pulse sequence (100 phases and 5 vps) on the respiratory motion model. For all four test cases, the respiratory model consisted of only one layer of isochromats in the slice selection direction. All gradients along the slice direction were turned off so that spurious echoes could only be potentially induced by the gradient crushers along the remaining directions. In the first three test cases real crushers were introduced along the readout direction at the end of each TR. The crushers had duration of 0.5 msec and areas equal to the area of the readout gradient. For the first test case, the isochromat volume size was set to 0.5 mm × 0.5 mm × 1 mm (total number of isochromats: 10000). For the second test case, the isochromat volume size was set to 0.25 mm × 0.25 mm × 1 mm (total number of isochromats: 40000). For the third case, the isochromat volume size was set to 0.1 mm × 0.1 mm × 1 mm (total number of isochromats: 250000). This case represented the maximum isochromat volume in order to avoid the formation of spurious echoes, thus resulting in the minimum possible computational load for real crushers. For the fourth test case, software crushers were used instead of real crushers with an isochromat volume size equal to that of the first test case (0.5 mm × 0.5 mm × 1 mm, total number of isochromats: 10000). After validating the use of software crushers with the aforementioned four test cases, software crushers were used for all remaining experiments described herein.

**Figure 4:**
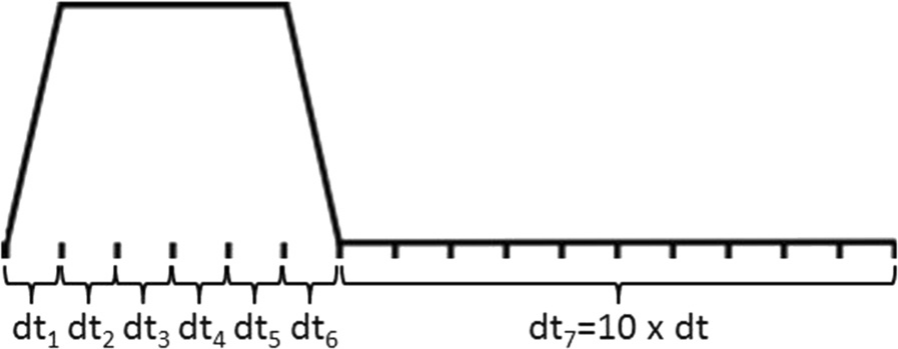
**Utilization of variable time step size.** Prolongation of the time step size can be done 1. when no temporal changes occur within the pulse sequence, 2. when the acquisition window is off, 3. when no pulses (RF and gradient) are on and 4. when no magnetic field inhomogeneities are considered.

Furthermore, as described in the previous study [17], MRISIMUL utilized an algorithm (“fast algorithm”) that allowed for a variable time step size during times when no changes occurred within the pulse sequence and when the acquisition window was off. For MR simulations involving motion models, the “fast algorithm” was updated so that longer time steps could be used when no RF and no static field gradients were applied. Figure 4 illustrates how the variable time step size was implemented in MR simulations that involve motion. As an example, the variable time step “fast algorithm” was applied for Gradient Echo and CINE pulse sequences with respiratory motion so as to measure the potential speedup gain.

**Figure 5:**
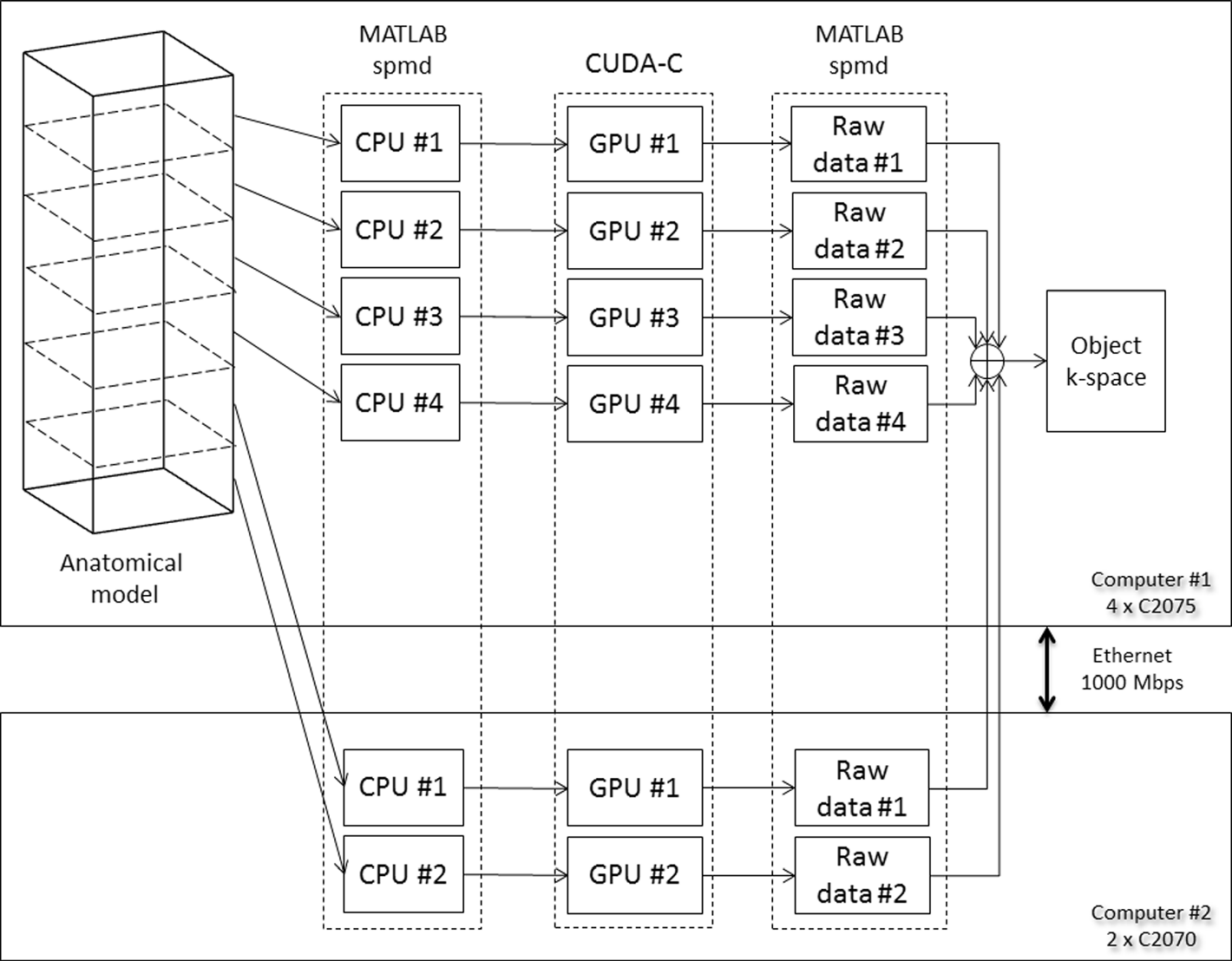
**Block diagram of the multi-node, multi-GPU approach.** One of the two computers was set as a job manager. Each CPU core was assigned a part of the anatomical model and a certain GPU-card. A balanced load among the GPUs was implemented.

Finally, for educational purposes, where speed is needed, the problem complexity should be limited to two dimensions (including a 2D motion model). For this purpose, as an example, the Gradient Echo pulse sequence simulation with respiratory motion was confined to two dimensions and the kernel execution times were recorded with and without the variable time step “fast algorithm”.

e. Parallel computing, mutli-node and multi-GPU systemsExperiments were performed on two different computer systems. The first was a single-node system consisting of a server style computer of 2 hexa-core (Intel E5-2630, 2.30 GHz) processors, 32 GB RAM and four Tesla C2075 GPU cards. The second was a multi-node system which included the aforementioned server as well as a desktop computer with 2 quad-core processors (Intel E5520, 2.27 GHz), 48GB RAM and two Tesla C2070 GPU boards. For this system, the two computers were connected via 1 Gbps Ethernet within the same subnet, resulting in a total number of 6 available GPU cards. One of the two single computers was set up as a job manager for distributing the computational load to the 6 available GPU cards and for reconstructing the total k-space and image. Figure 5 shows the block diagram for both single-node and multi-node system architectures.

Both C2075 and C2070 GPU boards share the same architecture: global memory of 6GB GDDR5 and 448 GPU cores grouped into 14 streaming multiprocessors of 32 processors each. The Tesla C2070 was based on the NVIDIA Tesla T20 graphics processing unit whereas the Tesla C2075 was based on the NVIDIA Tesla T20A graphics processing unit [26,27]. Both GPU cards had a compute capability of 2.0 and were targeted for high performance computing.

A benchmarking test was performed so as to ascertain the optimum combination of blocks and threads per block. For this test, the NVIDIA Visual profiler [28] was used and the kernel execution time was recorded for the application of a pulse sequence of 220800 time steps on a 3D object of 500000 isochromats. The number of blocks was always selected to be an integer multiple of the 14 streaming multiprocessors (available on both GPU card models) whereas the number of threads per block was always selected based on the available shared memory size per block [17]. Since the two different GPU cards models (C2070 and C2075) shared the same architecture, the resulting combination of blocks and threads per block was kept for all the experiments throughout this paper.

To demonstrate the efficacy of MRISIMUL on single-node and multi-node multi-GPU systems, MRISIMUL now supported the MATLAB single-program-multiple-data (spmd) statement. The total number of the available GPU boards in a system defined the processes spawned, each one accessing a different CPU. A GPU board along with a part of the anatomical object were assigned to each process and, in the end, the outputs of each process were summed together so as to form the total k-space matrix of the object. A balanced load among the GPUs was implemented.

**Figure 6:**
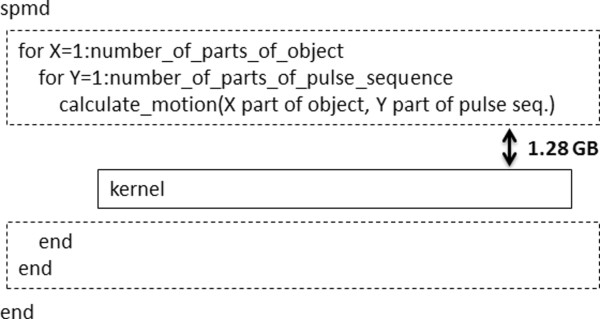
**Non-kernel embedded motion models algorithm.** The object was divided in X parts and the pulse sequence in Y parts. Every kernel call was accompanied by a transfer of 1.28GB of data to the GPU global memory through the high-speed serial computer expansion bus (PCIe ×16 Gen2, speed rate of 8GBs/sec).

To test for scalability in multi-GPU experiments, the execution times of the computational kernel were recorded for a total of 1, 2, 3, 4, 5 and 6 GPU cards. For these tests, a GE pulse sequence with the respiratory motion model was used. The total number of time steps was 640000. The anatomical computer model consisted of 2000000 isochromats.

f. Non-kernel embedded motion modelsThe motion models described by equations (5) through (10) were embedded in the design of the CUDA kernel as previously described. However, motion models may not always be available in an analytical expression, for example, particle flow measure recorded from patients. By definition, such measurements cannot be embedded in the design of the CUDA kernel. Even when analytical expressions of motion are available, in many cases it may be desirable not to redesign the CUDA kernel in order to embed a particular analytical motion expression to it. In these cases, the motion model may become a separate input to the kernel as a “non-predefined motion model”. In these cases, the motion model would need to be loaded or calculated directly on the host CPU prior to its transfer to the global memory of the GPU. Such non-predefined motion models were also tested. This approach required large amounts of memory space in both CPU and GPU since motion (i.e. the position of all the isochromats within the 3D anatomical object) needed to be saved for every time step of the pulse sequence. The limited memory space in both CPU and GPU required that the anatomical object and the pulse sequence be divided and that the kernel be called multiple times more than with the use of kernel embedded motion models (Figure 6).To evaluate the performance of non-predefined motion models, the respiratory motion model was considered as a “non-predefined motion model” whose temporal evolution, rather than being embedded in the kernel, was calculated directly on the host CPU prior to its transfer to the global memory of the GPU. This allowed for direct comparisons of execution times against the embedded model of the GE pulse sequence described earlier. This non-predefined motion model resulted in a total number of 640000 timesteps, 2000000 isochromats and required approximately 4 TB of total memory space. Since the current MATLAB and GPU configuration allowed transfer of matrices with size less than 2GB, the object was divided in 36 parts and the pulse sequence in 80 parts of 8000 timesteps each. The total experiment size was thus divided 36*80 = 2880 times. Thus, the CUDA kernel was called 2880 times. For each kernel call, a dataset of 1.28GBs (Figure 6) was transferred from the host to the GPU through a high-speed serial computer expansion bus (PCIe ×16 Gen2, speed rate of 8GBs/sec). The total execution time of the kernel was recorded for different combinations of the available GPU cards on a single-node computer system.

In this study, no experiment was carried out on humans or on animals.

## Results

### Benchmarking results

The kernel execution times, for the 24 different pairs of blocks and threads per block, are shown in Table 1, which identifies the optimum combination of blocks and threads. For the current design of the algorithm, the shorter kernel execution time was achieved for 112 blocks and 128 threads per block therefore resulting in simulating a total of 14336 isochromats per kernel call.

**Table 1:** Kernel performance was assessed for different combinations of blocks and threads/block

**Threads/Block**	**64**						**128**					
**Blocks**	14	28	56	112	224	448	14	28	56	**112**	224	448
**Kernel time (sec)**	154.6	82.3	47.0	33.5	33.4	37.2	87.1	51.6	32.9	32.8	36.6	43.8
**Threads/Block**	**256**						**512**					
**Blocks**	14	28	56	112	224	448	14	28	56	112	224	448
**Kernel time (sec)**	64.0	33.4	33.5	37.4	44.7	59.1	34.8	34.5	38.9	46.3	61.5	60.1

Kernel execution times were compared among different combinations of blocks and threads/block for the application of a pulse sequence of 296000 time steps on a 3D object of 500000 isochromats. The shortest kernel execution time has been achieved for 112 blocks and 128 threads per block (shown in bold letters).

### Respiratory motion

**Figure 7:**
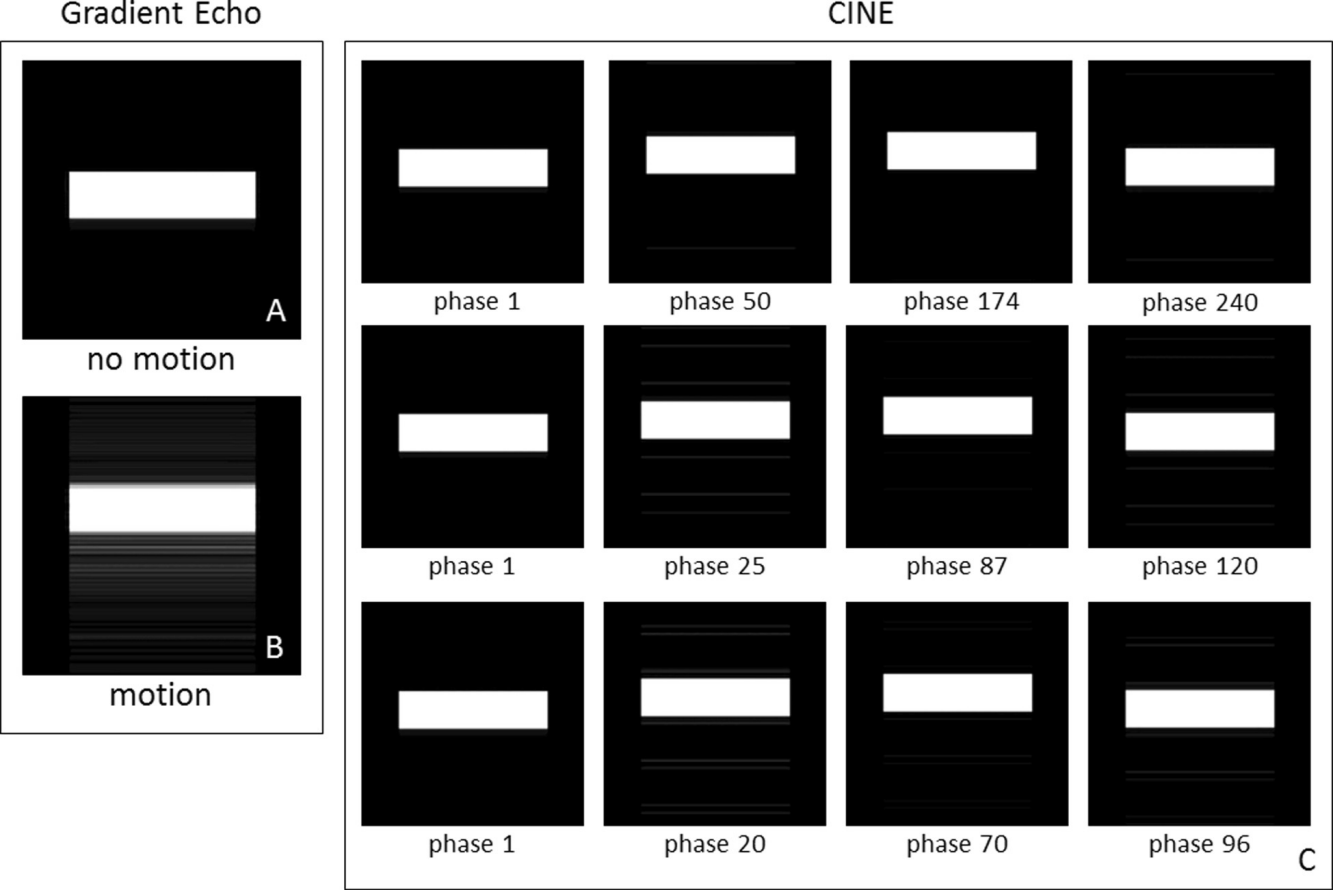
**GE images of the respiratory motion model. (A)** Application of the gradient echo pulse sequence under no motion. **(B)** Application of the gradient echo pulse sequence on the respiratory motion model without triggering. **(C)** Application of the CINE GE pulse sequence with triggering on the respiratory motion model for different combinations of phases per cycle and views per segment: 250 phases and 2 views per segment (1^st^ row), 125 phases and 4 views per segment (2^nd^ row) and 100 phases and 5 views per segment (3^rd^ row). (Total number of tissue isochromats 2000000, total number of time-steps for GE: 1280000, for CINE 1^st^ row: 55200000, for CINE 2^nd^ row: 27600000, for CINE 3^rd^ row: 22400000, k-space matrix 256 × 256, FOV 15 cm × 15 cm, slice thickness 10 mm, kernel execution time on the 4×C2075 system for GE: 8 min, for CINE 1^st^ row: 518 min, for CINE 2^nd^ row: 258 min, for CINE 3^rd^ row: 213 min). The entire 3D object was simulated; not just the slice of interest. Contrast has been exaggerated to visualize the residual artifact in the noise floor.

Figure 7 (left column) shows GE images obtained with the simulator both without motion (A) and with the respiratory motion model (B). Since this GE pulse sequence is not triggered to the respiratory motion (B) a motion artifact appears in the phase encoding direction. Figure 7 also shows CINE GE images triggered to the respiratory motion model for different combinations of phases per cycle and views per segment. Figure 7C illustrates different phases during the respiratory cycle for 250 phases and 2 views per segment (1^st^ row), 125 phases and 4 views per segment (2^nd^ row) and 100 phases and 5 views per segment (3^rd^ row). For a respiratory model displacement of 12 mm, the measured displacement from the CINE GE images was equal to 12.31 ± 0.03 mm.

The implementation of the variable step size “fast algorithm” with the Gradient Echo pulse sequence experiment provided a speedup of four times. The CINE pulse sequence did not benefit at all from the “fast algorithm”. In terms of accuracy, the Gradient Echo pulse sequences were compared with and without the variable step size “fast algorithm”. For k-space data, the difference between the two was on the order of 0.1% [29]. Finally, for educational purposes, the Gradient Echo pulse sequence experiment was limited to two dimensions (including a 2D motion model) resulting to a total number of 10000 isochromats. This educational 2D configuration demonstrated a kernel execution time of 3.9 sec (speedup of 123 times relative to the 3D configuration, which run in 8 mins) when the variable step size “fast algorithm” was off. When the variable step size “fast algorithm” was on then the 2D configuration run the kernel in 3.7 sec (speedup of 32 times relative to the 3D configuration, which run in 2 mins).

### Heart motion

**Figure 8:**
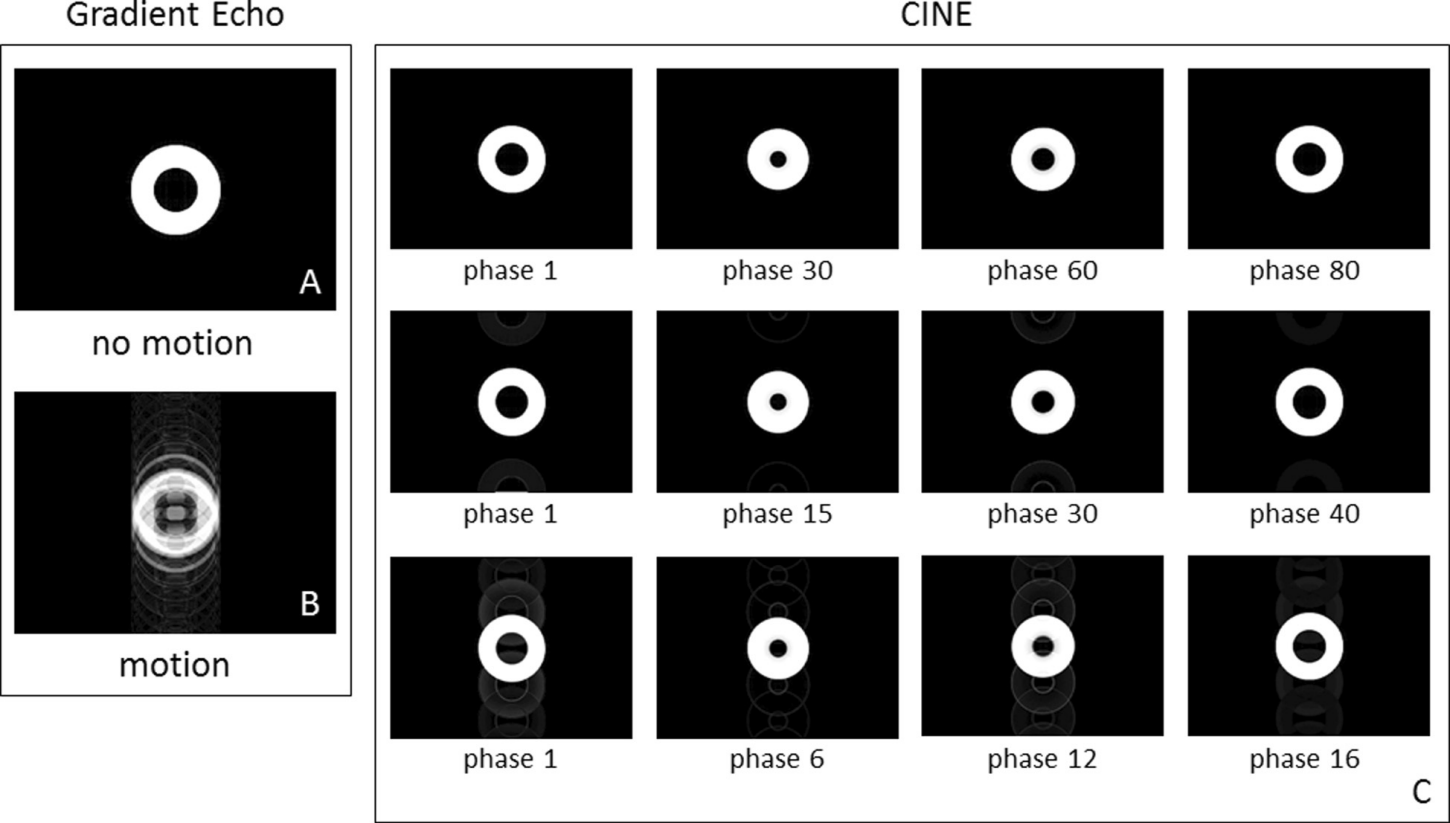
**GE images of the heart motion model. (A)** Application of the gradient echo pulse sequence on the heart model under no motion. No artifacts observed. **(B)** Application of the gradient echo pulse sequence on the heart motion model with no triggering. Phase direction artifacts are observed. **(C)** Application of the CINE GE pulse sequence triggered to the heart motion model for different combinations of phases per cycle and views per segment: 100 phases and 1 view per segment (1^st^ row), 50 phases and 2 views per segment (2^nd^ row) and 20 phases and 5 views per segment (3^rd^ row). (Total number of tissue isochromats 7632792, total number of time-steps for GE: 1280000, for CINE 1^st^ row: 22080000, for CINE 2^nd^ row: 11040000, for CINE 3^rd^ row: 4480000, k-space matrix 256 × 256, FOV 36 cm × 27 cm, slice thickness 10 mm, kernel execution time on the 4×C2075 system for GE: 36 min, for CINE 1^st^ row: 887 min, for CINE 2^nd^ row: 434 min, for CINE 3^rd^ row: 175 min). The entire 3D object was simulated; not just the slice of interest. Contrast has been exaggerated to visualize the residual artifact in the noise floor.

**Figure 9:**
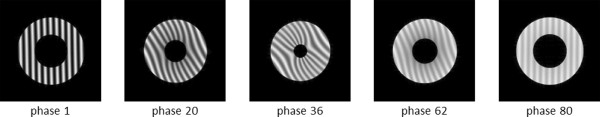
**Series of images of the heart model during motion, obtained after a 1–1 SPAMM preparation, producing saturation bands.** Note the gradual loss of contrast between the bands and myocardium throughout the cardiac cycle due to T1. Kernel execution time on the 4×C2075 system: approximately 7 hours. The entire 3D object was simulated; not just the slice of interest. (Total number of tissue isochromats 7 632 792, total number of time-steps 11 943 600, k-space matrix 128 × 128, FOV 15 cm × 15 cm, slice thickness 10 mm, 100 phases per cardiac cycle and 1 view per segment).

Figure 8 (left column) shows the results obtained with MRISIMUL and the application of the GE pulse sequence on the heart motion model. GE images were acquired in the absence of ECG triggering from the heart anatomical model both without motion (A) and with motion (B). The motion artifact caused by the application of non-triggered GE pulse sequence is seen in (B) along the phase encoding direction. Figure 8 (right table) also shows CINE GE images triggered to the motion of the anatomical heart model for a variety of combinations of phases per cycle and views per segment, as mentioned in Methods. Figures 8C illustrates different phases during the cardiac cycle for 100 phases and 1 view per segment (1^st^ row), 50 phases and 2 views per segment (2^nd^ row) and 20 phases and 5 views per segment (3^rd^ row). As expected, a small ghosting artifact is present in phases where the object is moving and multiple k-space lines are acquired per segment (such as Figure 8C, phase 12, 3^rd^ row). The inner radius of the heart model (24 mm undeformed and 10 mm deformed) was measured from the CINE GE images to be equal to 24.4 ± 0.52 mm undeformed and equal to 10 ± 0.13 mm deformed.Last, the ability of MRISIMUL to perform myocardial tagging is displayed in Figure 9. This demonstrates the temporal evolution of the contractility of the heart model based on the tagging that has been applied along the readout direction. Contrast reduction due to T1 relaxation can also be observed throughout the cardiac cycle.

### Flow motion

**Figure 10:**
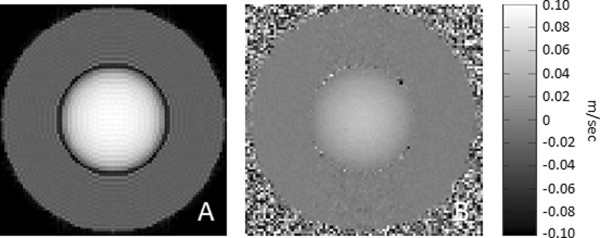
**Images obtained by applying a PC velocity encoded GE pulse sequence on the flow model. (A)** Magnitude image obtained from the flow model. **(B)** Corresponding phase contrast velocity image. The entire 3D object was simulated; not just the slice of interest. (Total number of tissue isochromats 30779693, total number of time-steps 1380000, k-space matrix 256 × 256, slice thickness 10 mm, Kernel execution time on the 4×C2075 system: 111 min for each experiment).

Figure 10 shows results obtained from the MRISIMUL simulator by applying a Phase Contrast (PC) velocity encoded gradient echo pulse sequence on the flow model described earlier. Figure 10A depicts the magnitude image obtained from one of the velocity encoded experiments whereas Figure 10B depicts the phase contrast velocity image. Stationary tissue is depicted in mid-grey whereas flowing spins are depicted in shades of mid-gray to white. The maximum velocity was measured from the phase contrast velocity images and found to be in good agreement with the heart model (model maximum velocity 5 cm/s, measured velocity 5.08 ± 0.14 cm/s).

### Pulse sequence design and computational demands

**Figure 11:**
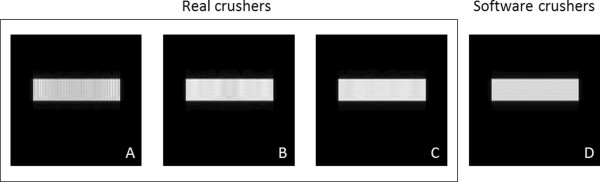
**Gradient crusher induced artifacts can be eliminated with software crushers.** In images A-C real crushers have been used. **(A)** shows an image with a fast execution time kernel (6 min) with a stripe pattern artifact related to low isochromat density (volume size: 0.5 mm × 0.5 mm × 1 mm, total number of tissue isochromats: 10000) **(B)** increasing medium isochromat density reduces the stripe artifact (volume size: 0.25 mm × 0.25 mm × 1 mm, total number of tissue isochromats: 40000, Kernel execution time: 13 min) **(C)** Using a high isochromats density eliminates the artifact at the expense of prolonged execution times (isochromat volume size: 0.1 mm × 0.1 mm × 1 mm, total number of tissue isochromats: 250000, Kernel execution time: 60 min) **(D)** With software gradient crushers (all other parameters as in A) and low isochromats density the artifact is not visible (Kernel execution time: 6 min). All experiments were run on a 4×C2075 system.

MRISIMUL allows the introduction of two forms of crushers, either in the form of gradient pulses or as software crushers. Figure 11 shows how software crushers may result in shorter execution times and may better utilize GPU resources. With CINE GE, it can be seen that a lower isochromat density (Figure 11A) might induce a phase difference of more than 180° among neighboring isochromats. Notice the resulting stripe pattern artifact. This artifact was not present when software crushers were implemented and the spatial density remained unchanged (Figure 11D).

### Parallel computing, mutli-node and multi-GPU systems

**Figure 12:**
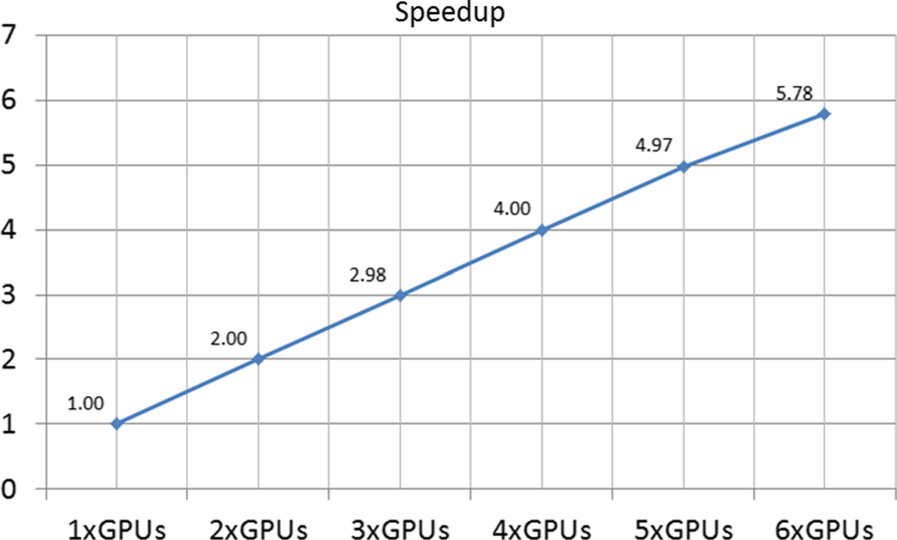
**Speedup observed with increasing number of available GPU cards on a multi-node, multi-GPU computer system.** Performance is normalized to the single GPU board system, which is the reference standard for all comparisons. An almost linear dependence is observed. (Kernel execution time: 16 min for 1×GPU, 8 min for 2×GPUs, 5.36 min for 3×GPUs, 4 min for 4×GPUs, 3.22 min for 5×GPUs, 2.76 min for 6×GPUs).

The execution times of MRISIMUL’s kernel were recorded on both single-node computer systems and on a two node multi-GPU computer system. The performance of the simulator was evaluated with the GE pulse sequence on the respiratory motion model (Total number of tissue isochromats 500000, total number of time-steps: 640000) as mentioned earlier. Figure 12 demonstrates an almost linear scalable performance along with the increasing number of the available GPU cards on both single-node and two-node multi-GPU systems.

### Non-kernel embedded motion models

**Figure 13:**
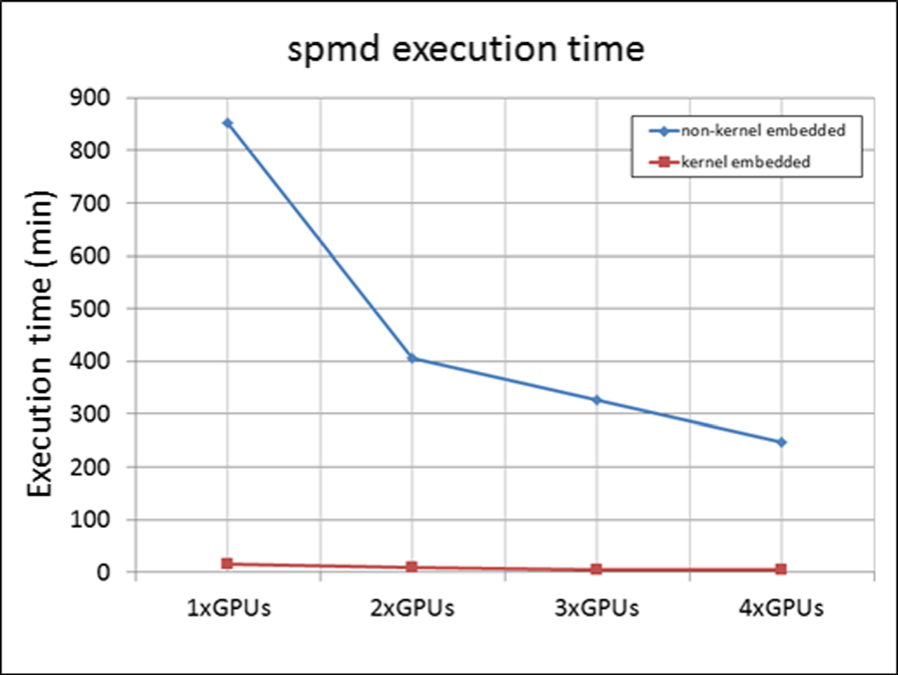
**Simulation of kernel embedded and non-kernel embedded motion models.** The blue line shows execution time of the spmd statement for simulating a non-kernel embedded motion model with an increasing number of GPUs on a single-node computer system. The red line shows the execution times for simulating the same motion model using a kernel embedded implementation with an increasing number of GPUs. While non-kernel embedded models take longer to execute, they allow for using non-analytically described motion patterns. (Total number of tissue isochromats 2000000, total number of time-steps 640000. Non-kernel embedded execution times: 851.4 min for 1×GPU, 405.5 min for 2×GPUs, 327.5 min for 3×GPUs, 248.3 min for 4×GPUs, Kernel embedded execution times: 16.4 min for 1×GPU, 8.3 min for 2×GPUs, 5.6 min for 3×GPUs, 4.3 min for 4×GPUs). All experiments were run on a 4×C2075 system.

Last, non-kernel embedded motion models were also investigated. While requiring longer execution times, they allow for more complex motion patterns that are not necessarily described analytically. The test case was a GE pulse sequence of 640000 timesteps on the respiratory motion model with 2000000 tissue isochromats. Figure 13, blue line, shows the total execution time of the spmd statement of the non-kernel embedded motion model as recorded for different combinations of the available GPU cards on a single-node computer system. As a reference, the execution of the kernel embedded motion model is in red.

## Discussion

A multi-GPU approach of the MRISIMUL simulator that supported simulations of general-purpose, realistic, motion-related MR experiments was presented in this study. MRISIMUL is the first MR physics simulator to have implemented motion with a 3D large computational load on a single computer multi-GPU configuration. The use of both single-node and multi-node multi-GPU systems allowed for an almost linear speedup with increasing number of GPU boards. Non-kernel embedded motion models, even though they take longer to execute, were successfully demonstrated as a solution to using motion models that cannot be described analytically. The use of software crushers was presented as an alternative to real crushers for allowing for further simulation speedup. The variable time step “fast algorithm” was also examined in this study so as to measure the potential speedup gain.

Three different motion models were simulated in this work, including a respiratory motion model, a heart motion model and a simple flow model. The respiratory motion model simulated one-directional motion of the diaphragm whereas the heart motion model simulated myocardial deformation due to longitudinal and radial contraction, axial torsion and rigid body displacement. Last, laminar flow of a homogeneous fluid within a straight tube was also simulated. Gradient Echo and CINE Gradient Echo pulse sequences were applied to these motion models so as to demonstrate motion-related artifacts and to explore motion-related MR applications.

Selecting the minimum number of isochromats, so as not to introduce spurious echoes, ensured the minimum execution time by the simulator. For non-moving isochromats the accrued phase difference among neighboring isochromats due to gradient fields was computed, prior to downloading the pulse sequence to the CUDA engine. The density of the isochromats was automatically adjusted so that this phase difference remained below 180 degrees [17]. For non-deforming moving tissue or laminar flow the relative distances between neighboring isochromats did not change and therefore the aforementioned scheme performed well. For deforming tissue the local strain was taken into account and the minimum acceptable distance between isochromats was further decreased according to the strain; then the aforementioned algorithm was applied. For more complex types of motion, such as turbulent flow, this solution no longer works. In this case, the temporal evolution of the motion model in 3D would have to be examined so as to limit the maximum phase to less than 180 degrees between neighboring moving isochromats. Also, certain restrictions would have to be imposed on the motion model so that isochromats cannot occupy the same spatial location. The current implementation of MRISIMUL does not address this issue.

In pulse sequence design, MRISIMUL introduced a second form of crushers, namely software crushers. These crushers induced nullification of the transverse components of every isochromat within the anatomical object. The replacement of real gradient crushers by software crushers allowed for not being forced to increase isochromat spatial density. This resulted in faster execution times without spurious echo formation. In simulations that involve real crushers and anatomical model deformations (such as stimulated echoes applied to the myocardium), the required spatial density of the model may not always be obvious due to tissue strain. Special considerations have to be made in such cases.

In this work, external Bo and B1 maps with time dependence were not studied. Also, spurious echoes, as described above, may arise when ΔBo is time dependent and unknown ahead of time thus resulting in suboptimal modeling of T2* when a low number of isochromats per voxel is used. Also, with MRISIMUL all fields and positions are assumed constant within each time step of the simulator thus setting a limit on the permissible length of the time step when considering fast motion. The maximum time step length is dependent on the spectral content of the motion. At the expense of execution time, the aforementioned problems can be mitigated by shorter time steps and increased number of isochromats.

Last, the simulator’s performance was evaluated on different computer systems, including single-node and multi-node multi-GPU computer systems. In order to accommodate the high computational load introduced by simulation of motion experiments, the kernel distributed in a balanced manner the computational load to multiple GPUs. MRISIMUL demonstrated an almost linear scalable performance with the increasing number of available GPU cards, in both single-node and multi-node multi-GPU computer systems.

In the past, two other advanced MR physics simulators have incorporated the study of motion in their simulations; namely POSSUM [10] and JEMRIS [16]. However, both of them presented the following limitations/assumptions: 1. they allowed only simulation of simple translational and/or rotational motion models and 2. simulations were usually limited within the slice of interest and did not include the entire 3D anatomical model. Although the latter assumption is not related to any specific design limitation of these simulation platforms, the main reason for imposing it was the high computational load introduced by the simulation of an entire motion model during the entire course of the pulse sequence. Compared to these simulators, MRISIMUL exploited the high computational power of GPU technology and allowed for simulating realistic motion models in the entire 3D anatomical object during the entire course of the MR pulse sequence. Moreover, the high computational power of MRISIMUL further enhanced the utilization of non-kernel embedded motion models that could not be simulated within the GPU kernel in an analytical manner.

## Conclusion

In conclusion, MRISIMUL is an MR physics simulator that allows for computationally intense simulations of general-purpose, realistic, 3D motion-related MR experiments on single-node computer systems by taking advantage of the high computational power of multi-GPU configurations. The incorporation of realistic motion models, such as heart motion and flow models may benefit the design and optimization of existing or new MR pulse sequences, protocols and algorithms that examine motion related MR applications. While further development of more advanced motion models is under way and validation of them against well-established clinical protocols is warranted in the future, one should note that the development of an MRI simulator that incorporates computationally demanding motion models would not have been possible only a few years ago due to lack of appropriate GPU hardware.

An online version of MRISIMUL is available through http://mri.dib.uth.gr along with the pulse sequences presented in this study.

## Competing interests

The authors declare that they have no competing interests.

## Authors’ contributions

CX and AA carried out the experiments design. CX carried out the simulator development and data collection. CX and IV carried out the refinement of the simulator kernel and the collection of the benchmarking results. All authors read and approved the final manuscript.
